# Maternal mental health and coping during the COVID‐19 lockdown in the UK: Data from the COVID‐19 New Mum Study

**DOI:** 10.1002/ijgo.13397

**Published:** 2020-10-16

**Authors:** Sarah Dib, Emeline Rougeaux, Adriana Vázquez‐Vázquez, Jonathan C. K. Wells, Mary Fewtrell

**Affiliations:** ^1^ UCL Great Ormond Street Institute of Child Health London UK

**Keywords:** Coping, Coronavirus, COVID‐19, Maternal health, Mental health, Postpartum

## Abstract

**Objective:**

To assess how mothers are feeling and coping during lockdown, and to identify the potential pathways that can assist them.

**Methods:**

A descriptive analysis of maternal mental health, coping, support, activities, lockdown consequences was conducted. Women living in the UK with an infant aged ≤12 months completed an online survey. Linear regression was used to identify predictors of maternal mental health and coping.

**Results:**

A majority of the 1329 participants reported feeling down (56%), lonely (59%), irritable (62%), and worried (71%) to some extent since lockdown began, but 70% felt able to cope. Support with her own health (95% confidence interval [CI] 0.004–0.235), contacting infant support groups (95% CI −0.003 to 0.252), and higher gestational age of the infant (95% CI 0.000–0.063) predicted better mental health. Travelling for work (95% CI −0.680 to −0.121), the impact of lockdown on the ability to afford food (95% CI −1.202 to −0.177), and having an income <£30 000 (95% CI −0.475 to −0.042) predicted poorer mental health. Support with her own health and more equal division of household chores were associated with better coping.

**Conclusion:**

There is a need to assess maternal mental health and identify prevention strategies for mothers during lockdown.

## Abstract 2

During lockdown, many new mothers experienced symptoms of poor mental health; urgent action is needed to support postpartum mothers during the pandemic and beyond.

## 1 INTRODUCTION

The coronavirus disease 2019 (COVID‐19) pandemic resulted in a rapid change of circumstances in the UK population, and many individuals may have experienced loss of livelihood, increased financial burden, reduced personal support systems and professional services, physical isolation, and illness. Therefore, mental health is likely to be affected, as is already evident. A recent paper published in *The Lancet* has highlighted the urgent need for research that tackles how the effects of COVID‐19 on mental health could be eased, especially in vulnerable populations.^1^

These effects may be of particular importance for mothers during pregnancy and in the first year after giving birth. According to data from before the pandemic, perinatal mental illness affects up to 20% of new and expectant mothers, and is associated with an increased risk of preterm delivery, reduced mother–infant bonding, and decreased odds of breastfeeding.^2^, ^3^ It is expected that this rate has increased^4^ as a result of physical and social isolation, changes in perinatal services, and the economic burden of the disease that is disproportionately affecting women.^5^, ^6^ Several studies have highlighted the increase in distress and psychological problems experienced by pregnant women^7^, ^8^, ^9^ and postpartum women during the pandemic.^10^, ^11^ In the UK, it was recently reported that new mothers with babies aged under 1 year expressed feelings of being robbed of the joys of motherhood,^12^ which was also noted in a recent qualitative study.^13^

The aim of the present study was to provide descriptive data on maternal mental health, coping, support, and activities during week 1 of the study (May 27 to June 3, 2020). Possible pathways for the effects of the COVID‐19 lockdown on maternal mental health and coping are also investigated.

## 2 MATERIALS AND METHODS

Women living in the UK aged 18 years or older who have an infant currently under 12 months of age are being invited to complete a one‐time, anonymous online survey. Information and links to the survey are shared on Facebook, Twitter, and Instagram pages and groups used by mothers such as infant feeding (breastfeeding, bottle feeding, formula feeding) support, parent/mother/women’s support and neighborhood/town/city groups. It is also being shared via relevant professional groups and contacts, and via word of mouth. The survey, which uses the professional software RedCap, was launched on May 27, 2020, and will remain open until at least December 31, 2020, to capture data relating to the different levels of lockdown restrictions.

The full content of the survey, which includes details about the background factors, infant feeding practices, and impacts of COVID‐19, has been described elsewhere.^12^ For the present analysis, data were collected regarding:

1. Background characteristics: social and demographic factors
2. Consequences of lockdown: advised to shield/isolate, gave birth before or during lockdown, and impact of lockdown on the family’s ability to afford rent, food, and other essentials.
3. Mother’s activities, access to support, and perceptions: questions asked about how often (0 times, 1–3 times, 4–5 times, or daily) the participants engaged in activities in the previous week, such as walking, exercise, relaxation techniques, and grocery shopping. Frequency of accessing infant support groups and contact with healthcare and mental health professionals was also collected to assess the level of support received. Maternal perceptions of lockdown were assessed retrospectively, where participants were asked to state how much the following statements applied to them since lockdown began (not at all, very little, to some extent, to a high extent): I’ve been feeling down; I've been feeling lonely; I've had trouble relaxing; I've become easily annoyed or irritable; and I've been feeling worried. Appetite and disruption to sleep were also similarly assessed. We also included “positive” statements, including: I feel able to cope with the situation; I’ve enjoyed the spring weather; I’ve had the opportunity to chat with my family and friends; I’ve had time to enjoy personal interests or hobbies; I’ve had time to focus on my health; and I’ve had time to exercise.

Since this is an anonymous survey and the participants cannot be identified for follow‐up, no formal assessment of depression or anxiety (using validated tools) was undertaken. Participants were also given the option to omit any sensitive questions they did not wish to answer. A list of resources for infant feeding and maternal mental health support, including resources from organizations providing services specifically to people belonging to Black, Asian, and Minority Ethnic (BAME), LGBTQ+, and disabled groups, is provided at the end of the survey.

Ethical approval was obtained from the UCL Research Ethics committee (0326/017). The first page of the survey provides information about the study and, having read this, participants were asked to provide consent to participate before proceeding.

**Figure 1:**
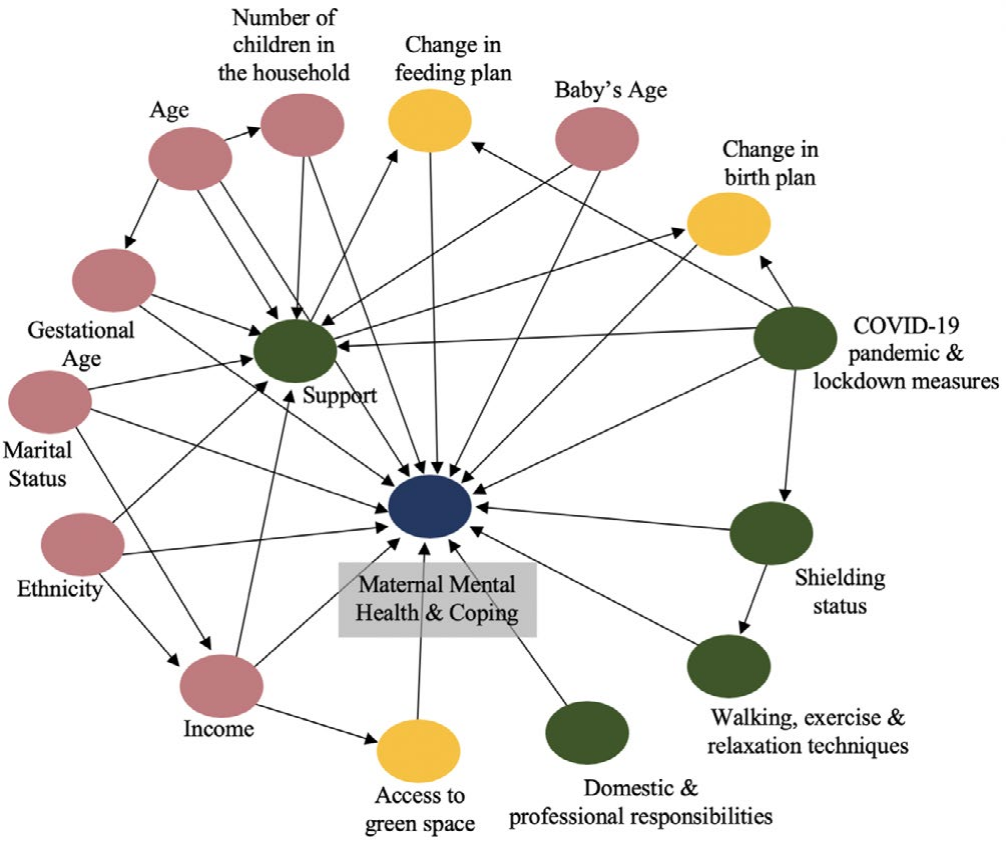
Directed acyclic graph of the relationship between maternal mental health or coping and activities, support and consequences of lockdown.

Data from the survey were exported from RedCap, the software collecting survey responses, and analyzed in SPSS version 26 (IBM., Armonk, NY, USA). Descriptive data are shown as number (percentage) or mean ± standard deviation. Descriptive characteristics of the population and description of maternal mental health, support received, activities undertaken, and consequences of lockdown were presented for the whole sample. Principal component analysis (PCA) was conducted for the maternal perceptions; Kaiser‐Meyer‐Olkin Measure of Sampling Adequacy and the Bartlett test of sphericity were used to assess whether PCA is appropriate. Eigenvalues (of 1), scree plots, and parallel analysis were used to identify the components of perceptions. Regression variables were saved, and then linear regression was conducted to assess the relationship between mental health and coping (outcomes) and support, activities, and consequences of lockdown (as predictors). Directed acyclic graphs (DAG) (Fig. 1) were sketched to identify the minimum adjustment set of confounders and to identify ancestors of the outcome that were dropped from the regression model. On this basis, the linear regression models were controlled for maternal age, baby’s age, ethnicity, gestational age, income, marital status, and number of children in the household. 95% confidence intervals (CI) are presented for all regression coefficients and *P* values less than 0.05 are considered statistically significant.

## 3 RESULTS

During the first week of the study, 1329 participants fully completed the survey, the majority of which self‐identified as white, were married or living with a partner, lived in a house, and had access to green space within walking distance (Table 1).

**Table 1:** Background characteristics of women who completed the survey in week 1.^a^

Maternal age (years)	31.7 ± 4.7
Infant age (months)	4.8 ± 3.1
Infant gestation (weeks)	39.3 ± 1.8
Male infant	675 (51)
Having other children in the household	601 (45.2)
Maternal ethnicity	
White	1251 (94.0)
Mixed/multiple ethnic groups	35 (2.7)
Black/African/Caribbean/black British	6 (0.5)
Asian/Asian British	28 (2.1)
Arab	1 (0.1)
Other ethnic group	3 (0.2)
Maternal education	
≤5 GCSE grade A–C	58 (4.4)
≥5 GCSE grade A–C	100 (7.5)
A levels/equivalent	280 (21.0)
Batchelor’s degree	521 (39.1)
Master’s degree	183 (13.7)
PhD/professional qualification	180 (13.5)
Living conditions	
Flat/apartment	173 (13.0)
House/bungalow	1142 (85.8)
Mobile/temporary structure	9 (0.7)
Access to green space within walking distance	1306 (98.1)
Marital status	
Married/Civil partnership/Cohabitation	1267 (95.2)
Single parent—living on own	34 (2.6)
Single parent—living with family	16 (1.2)
Household income	
<£20,000	87 (6.5)
<£30,000	139 (10.4)
<£45,000	238 (17.9)
<£75,000	428 (32.2)
<£100,000	200 (15.0)
>£100,000	179 (13.4)
Prefer not to say	53 (4.0)

^a^ Values are given as number (percentage) or mean ± standard deviation.

Four PCA components were identified from maternal perceptions (Table 2), which were labelled as follows: (1) “maternal mental health” (an indicator of low mood, anxiety, and loneliness, although not formally assessed); (2) “time availability”; (3) “coping”; (4) appetite and sleep changes (Table S1). In the present study, the main focus was maternal mental health and coping components as outcomes.

**Table 2:** The extent to which the participants agreed with the following perceptions, since the lockdown began.^a^

	Not at all	Very little	To some extent	To a high extent
Maternal mental health				
I’ve been feeling down	161 (12.1)	414 (31.1)	529 (39.7)	222 (16.7)
I’ve been feeling lonely	232 (17.4)	310 (23.3)	472 (35.5)	316 (23.7)
I’ve had trouble relaxing	220 (16.5)	336 (25.2)	465 (34.9)	301 (22.6)
I’ve become easily annoyed or irritable	143 (10.7)	354 (26.6)	502 (37.7)	328 (24.6)
I’ve been feeling worried	98 (7.4)	291 (21.9)	532 (40.0)	407 (30.6)
Coping				
I’ve had the opportunity to chat with my family and friends	4 (0.3)	100 (7.5)	602 (45.2)	619 (46.5)
I’ve enjoyed the spring weather	46 (3.5)	150 (11.3)	485 (36.4)	645 (48.5)
I feel able to cope with the situation	85 (6.4)	320 (24.0)	748 (56.2)	178 (13.4)
Changes to appetite and sleep				
I’ve had trouble falling or staying asleep	460 (34.6)	339 (25.5)	326 (24.5)	205 (15.4)
I’ve been having a poor appetite	848 (63.7)	230 (17.3)	196 (14.7)	49 (3.7)
Time to focus on health and interests				
I’ve had time to enjoy personal interests	635 (47.7)	476 (35.8)	169 (12.7)	48 (3.6)
I’ve had time to focus on my health	374 (28.1)	549 (41.2)	313 (23.5)	93 (7.0)
I’ve had time to exercise	284 (21.3)	481 (36.1)	391 (29.4)	173 (13.0)

^a^ Values are given as number (percentage).

More than half of the participants reported feeling down, lonely, and irritable, and had trouble relaxing, and 71% expressed feeling worried to some or to a high extent since the beginning of lockdown (Table 2). Despite these perceptions, the majority of the mothers expressed feeling able to cope with the situation (70%) and enjoy the weather (85%), and having the opportunity to connect with family and friends (92%) to some or to a high extent.

Table 3 shows the activities the participants engaged in during the previous week. The most commonly cited relaxation techniques were yoga, meditation, and breathing; 37% of mothers started using these methods during lockdown whereas the rest practiced relaxation techniques regularly before the lockdown. Fifty‐five mothers travelled to work at least once a week. Of these, 38% (n=21) worked in the NHS or medical/healthcare sector, followed by business/administration/accounting (n=9) and education (n=7).

**Table 3:** Activities the participants engaged in during the previous week.^a^

	0 times	1–3 times	4–5 times	Daily or more
Went shopping at the grocery store or pharmacy	625 (47.2)	670 (50.6)	25 (1.9)	4 (0.3)
Travelled for work	1269 (95.3)	44 (3.3)	4 (0.3)	7 (0.5)
Went outside for a walk or for exercise	67 (5.1)	438 (33.2)	296 (22.4)	520 (39.4)
Practiced a relaxation technique	1021 (76.7)	218 (16.4)	57 (4.3)	32 (2.4)

^a^ Values are given as number (percentage).

The different types of support received were assessed (Table 4). Only nine participants reported attending a face‐to‐face mother and baby or breastfeeding support group and four women had an in‐person session with a mental health professional, whereas the rest had contact online or by phone.

**Table 4:** Support measures for women who completed the survey in week 1.^a^

Got or getting enough support and help with own health	
Yes	783 (58.8)
No	545 (40.9)
Had contact with a mother and baby or breastfeeding support group^b^	
0 times	976 (73.3)
1–3 times	238 (17.9)
4–5 times	53 (4.0)
Daily or more	58 (4.4)
Had contact with a health professional (GP, health visitor, midwife)^b^	
0 times	791 (59.4)
1–3 times	496 (37.3)
4–5 times	31 (2.3)
Daily or more	5 (0.4)
Attended an appointment with a mental health professional^b^	
0 times	1236 (92.9)
1–3 times	81 (6.1)
4–5 times	10 (0.8)
Daily or more	1 (0.1)
I feel the household chores are more equally divided among household members	
Not at all	494 (37.1)
Very little	343 (25.8)
To some extent	298 (22.4)
To a high extent	186 (14.0)

Abbreviation: GP, general practitioner.

^a^ Values are given as number (percentage).

^b^ In the previous week.

For consequences of COVID‐19 and the lockdown, 27% of mothers delivered during the lockdown period, which has previously been shown to have had implications for birth and feeding plans. In addition, 8% were advised to isolate due to a pre‐existing health condition. Around one‐third expressed a minor to major impact of lockdown on the ability to pay rent or mortgage payments (37%), pay for food (32%), and pay for other essentials such as utilities and medication (28%).

Table 5 shows that getting enough support with the mother’s own health and contact with mother and baby support groups were predictors of better mental health. Mothers who reported that household chores were “only a little” more equally divided had better mental health than those who reported they were more equally divided “to a great extent.” Travelling for work, the impact of the COVID‐19 lockdown on the ability to afford food, and a lower household income (<£20,000–£30,000) were associated with worse mental health.

**Table 5:** Predictors of maternal mental health (anxiety, low mood, and loneliness).^a^

	**Variable**	**B**	**95% CI**	***P* value**
Contact in person, by phone, or online	Healthcare professionals^b^	−0.045	−0.167 to 0.077	0.470
	**Mother and baby support groups** ^b^	**0.124**	**−0.003 to 0.252**	**0.056**
	Mental health professional^b^	−0.197	−0.418 to 0.025	0.082
Extent to which chores are more equally divided^c^	**Enough help with own health**	**0.120**	**0.004–0.235**	**0.042**
	Not at all	0.101	−0.073	0.255
	**Very little**	**0.256**	**0.075–0.438**	**0.006**
	To some extent	0.091	−0.094 to 0.277	0.335
	Shopped at grocery store/pharmacy ^b^	0.063	−0.051 to 0.176	0.278
	Went outside for a walk or exercise ^b^	0.138	−0.109 to 0.385	0.274
	**Travelled for work** ^b^	**−0.401**	**−0.680 to −0.121**	**0.005**
	Practiced a relaxation technique^b^	0.113	−0.020 to 0.247	0.095
	Gave birth during lockdown	0.086	−0.082 to 0.254	0.313
Impact of lockdown on the ability to afford rent^d^ Impact of lockdown on the ability to afford food^d^ Impact of lockdown on the ability to afford other essentials^d^	Advised to shield due to risk	−0.045	−0.318 to 0.228	0.745
	Minor	0.075	−0.101 to 0.251	0.405
	Moderate	0.117	−0.110 to 0.345	0.312
	Major	0.063	−0.312 to 0.437	0.743
	Minor	0.170	−0.035 to 0.375	0.105
	Moderate	−0.204	−0.518 to 0.109	0.202
	**Major**	**−0.689**	**−1.202 to −0.177**	**0.008**
	Minor	−0.081	−0.313 to 0.151	0.494
	Moderate	0.193	−0.140 to 0.526	0.255
	Major	0.329	−0.234 to 0.891	0.252
Age	Maternal age	0.004	−0.009 to 0.017	0.525
	Infant’s age	0.017	−0.007 to 0.041	0.154
	**Infant’s gestational age**	**0.031**	**0.000–0.063**	**0.051**
Ethnicity^e^	BAME	−0.166	−0.398 to 0.066	0.160
Marital status^f^	Single—living alone	0.047	−0.311 to 0.405	0.798
	Single—living with family	−0.078	−0.598 to 0.443	0.770
Income^g^	**<£30,000**	**−0.258**	**−0.475 to −0.042**	**0.019**
	<£75,000	−0.045	−0.204 to 0.113	0.575
	<£100,000	−0.067	−0.260 to 0.126	0.496
Number of children in the household	1^h^	−0.005	−0.120 to 0.111	0.939

Abbreviations: BAME, black, Asian and minority ethnic group; CI, confidence interval; PCA, principal component analysis.

P values <0.05 were considered statistically significant and are given in bold.

^a^ PCA result of mental health. Higher scores reflect better mental health.

^b^ Participated in activities at least once in the previous week.

^c^ High extent as reference.

^d^ No impact as reference.

^e^ White ethnicity as reference.

^f^ Married/civil partnership/cohabitation as reference.

^g^ Income >£100,000 as reference.

^h^ More than one child as reference.

Table 6 shows that getting enough support with the mother’s own health predicted better coping. Conversely, with each unit decrease in the extent to which the participants perceived the household chores have become more equally divided, coping decreases.

**Table 6:** Predictors of coping.^a^

	Variable	B	95% CI	*P* value
Contact in person, by phone. or online	Healthcare professionals^b^	−0.101	−0.222 to 0.019	0.100
	Mother and baby support groups^b^	−0.092	−0.218 to 0.035	0.155
	Mental health professional^b^	−0.121	−0.340 to 0.099	0.281
Extent to which chores are more equally divided^c^	**Enough help with own health**	**0.388**	**0.274–0.503**	**0.000**
	**Not at all**	**−0.284**	**−0.456 to −0.113**	**0.001**
	**Very little**	**−0.245**	**−0.424 to −0.065**	**0.008**
	**To some extent**	**−0.216**	**−0.400 to −0.032**	**0.021**
	Shopped at grocery store/pharmacy^b^	0.044	−0.068 to 0.156	0.444
	Went outside for a walk or exercise^b^	−0.086	−0.330 to 0.159	0.493
	Travelled for work^b^	−0.199	−0.476 to 0.078	0.159
	Practiced a relaxation technique^b^	0.001	−0.131 to 0.133	0.990
	Gave birth during lockdown	0.031	−0.135 to 0.198	0.711
Impact of lockdown on the ability to afford rent^d^ Impact of lockdown on the ability to afford food^d^ Impact of lockdown on the ability to afford other essentials^d^	Advised to shield due to risk	−0.091	−0.362 to 0.179	0.508
	Minor	−0.033	−0.207 to 0.142	0.714
	Moderate	−0.061	−0.286 to 0.164	0.596
	Major	−0.002	−0.373 to 0.369	0.991
	Minor	−0.145	−0.348 to 0.058	0.162
	Moderate	−0.088	−0.399 to 0.223	0.578
	Major	−0.429	−0.936 to 0.079	0.098
	Minor	0.003	−0.227 to 0.233	0.980
	Moderate	0.215	−0.114 to 0.545	0.200
	Major	0.543	−0.014 to 1.099	0.056
Age	Maternal age	−0.003	−0.016 to 0.010	0.605
	Infant’s age	0.021	−0.003 to 0.044	0.089
	Infant’s gestational age	0.023	−0.008 to 0.054	0.142
Ethnicity^e^	BAME	−0.144	−0.374 to 0.085	0.218
Marital status^f^	Single—living alone	0.039	−0.315 to 0.394	0.827
	Single—living with family	0.220	−0.296 to 0.735	0.404
Income^g^	<£30,000	−0.185	−0.400 to 0.029	0.090
	<£75,000	−0.059	−0.216 to 0.098	0.459
	<£100,000	−0.007	−0.199 to 0.184	0.941
Number of children in the household	1^h^	0.039	−0.076 to 0.154	0.503

Abbreviations: BAME, black, Asian and minority ethnic group; CI, confidence.

interval; PCA, principal component analysis.

*P* values <0.05 were considered statistically significant and are given in bold.

^a^ PCA result of coping. Higher scores reflect better coping.

^b^ Participated in activities at least once in the previous week.

^c^ High extent as reference.

^d^ No impact as reference.

^e^ White ethnicity as reference.

^f^ Married/civil partnership/cohabitation as reference.

^g^ Income above 100K as reference.

^h^ More than one child as reference.

## 4 DISCUSSION

The findings of the present survey illustrate that despite the seemingly low‐risk population, a large proportion of new mothers reported symptoms of low mood, anxiety, and loneliness during the lockdown. Given the short‐ and long‐term consequences of perinatal mental illness on the physical and psychological well‐being of the mother and baby, there is an urgent need for action to support new mothers who have been affected.

It was found that the perception of how equal the division of chores among household members has been since lockdown began was associated with mental health and coping with the lockdown. In the present study, 63% of women felt that chores had become more equally divided since lockdown began. This is similar to the results of The Institute for Fiscal Studies,^6^ which showed that during lockdown, in two‐parent opposite‐gender families, fathers have doubled the time they spend on child care, but mothers still spent more time on child care and domestic responsibilities.

In the first year postpartum, women commonly experience a wide range of health problems such as tiredness, back pain, urinary incontinence, and more frequent minor illnesses. The association between physical health and mental health problems in the first year after childbirth is under‐researched; however, a few studies have highlighted the link.^14^ The present study provides further evidence that physical health should be an important target for interventions aiming to improve maternal mental health, as getting enough support with their own health was associated with better mental health and coping. Additionally, 69% of mothers in the present study reported having no or very little time during lockdown to take care of their own health. This emphasizes the role of partners, family, and social networks in helping mothers postpartum.

It was also found that support for mothers in the form of mother–baby or breastfeeding support groups was associated with better mental health (95% CI −0.003 to 0.252), while contact with healthcare professionals was not. This could be due to the type of support received (peer, professional) and the method by which it was given (remote, in‐person). Mother and baby support groups may be particularly important for peer support, where other mothers in these groups can relate to the experience of motherhood during lockdown and provide empathetic support and validation.^15^ Health professionals, instead, are likely to provide practical support, and due to restrictions, the majority of the contact with the mothers would have been by phone or online, which makes practical help more difficult. It has previously been reported that participants in this survey described their inability to access face‐to‐face practical support as a reason for changing their method of infant feeding.^12^ Health visitors have also voiced concerns over the reduced face‐to‐face home visits during lockdown, with 92% reporting parental mental health as a reason for the concern.^16^

The present study highlighted that travelling to work at least once in the previous week predicted worse mental health, with the most commonly reported jobs relating to the healthcare sector. Data from previous pandemics and from this pandemic in China and Italy is consistent with this, where 50.3% and 44.6% of healthcare workers reported experiencing depression and anxiety, respectively.^17^, ^18^ The higher risk of mental health problems for frontline workers during the pandemic, and for women in the postpartum period in general, suggests that these women are at particular risk.

Previous studies have shown that BAME groups, especially black women, and disadvantaged groups are disproportionately affected by COVID‐19.^19^, ^20^ The results from the present study did not show that ethnicity was associated with mental health or coping, probably due to the small sample size for the non‐white groups, but they indicated that having a lower income (<£20,000–£30,000) and the financial impact of COVID‐19 predicted worse mental health. It is also important to consider the quality of the housing, particularly its ability to provide adequate personal and outdoor space for household members. For example, 12% of households in Great Britain do not have access to a private or shared garden, and black people in England are four times less likely than white people to have access to outdoor space.^21^ These inequalities partially explain the variation in mental health outcomes among different groups. The majority of participants in the present study self‐identified as white, were married or living with a partner, lived in a house, and had access to green space within walking distance. This highlights the need to capture data from a more ethnically and socioeconomically diverse group of women.

Data from before the pandemic underlined that mothers of preterm infants experience higher stress, anxiety, and depression.^22^, ^23^ The lockdown measures, resulting in a decrease in support networks, changes in hospital policies, and concern about COVID‐19 infection, are likely to have exacerbated the mental health problems experienced by those mothers. The results of the present study suggest that a lower gestational age was associated with worse maternal mental health. Therefore, more attention should be given to this group.

Despite the high proportion of new mothers reporting symptoms of low mood, anxiety, and loneliness, a high proportion also indicated being able to cope with the situation. In this study, going outside for physical activity and relaxation techniques were investigated as potential methods used by the participants to cope with the lockdown. A previous study has shown that pregnant women engaging in at least 150 minutes of physical activity each week during the lockdown had lower anxiety and depression scores.^10^ No association was found between going out for physical activity and maternal mental health; however, the relatively uniform high levels of activity in the present study reduce the ability to detect an association between variability in level of activity and mental health or coping. As for relaxation techniques, a survey of 5545 Spanish adults during the lockdown found that relaxing activities (such as yoga, gardening, and listening to music) were associated with lower symptoms of anxiety and depression but only before correction for multiple comparisons.^24^ Similarly, in the present study, practicing relaxation techniques did not predict coping or mental health, possibly due to the variation in frequency, duration, and type of activities. Given the potential benefit of relaxation techniques and physical activity on anxiety and depression, further research is warranted.

With at least 98% of adults (16–45 years) in the UK accessing the Internet at least once a day, the online nature of this survey allowed for the rapid and remote capture of the experiences of mothers during the pandemic and made it possible to reach mothers from a wide geographical region. However, the use of social media might have resulted in biases in sampling: for example, women may not be part of the relevant social media groups or may have missed the links that were shared. It is also possible that this method has contributed to the underrepresentation of BAME and low‐income mothers (<£20,000–£30,000) in this sample, which is the main limitation of the present study. To tackle this, more emphasis is being placed on sharing the survey by word of mouth as well as collaborations with other organizations to reduce the reliance on social media. Another limitation is that it was not possible to compare the rates of depression or anxiety in new mothers during lockdown with the rates before the pandemic as mental illness was not formally assessed. However, a larger proportion of the general population do not fit the diagnoses of depression or anxiety, but are rather at risk, which makes the present results more generalizable. Changes in ‘symptoms’ of mental health and coping over the different phases of lockdown will also be monitored.

The results of this survey indicate that a large proportion of new mothers in the UK are experiencing symptoms of low mood, anxiety, and loneliness; mothers of preterm infants, those on a low income, and those who travel to work are particularly at risk. However, the data also suggest that providing support for the mother’s own health and with household chores are beneficial for maternal mental health and coping. Overall, the findings highlight the urgent need to assess maternal mental health and to create prevention strategies for mothers who are giving birth during the different stages of lockdown during this COVID‐19 pandemic.

## AUTHOR CONTRIBUTIONS

All authors contributed to the design and planning of the study. SD, MF, and JW contributed to the data analysis. SD drafted the manuscript. All authors read and approved the final manuscript.

## CONFLICTS OF INTEREST

The authors have no conflicts of interest.

## Supporting information


**Table S1.** Principal component analysis: rotated component matrix.
