# Supplementary material for: Maternal mental health and coping during the COVID‐19 lockdown in the UK: Data from the COVID‐19 New Mum Study
Source: Int J Gynaecol Obstet. 2020 Oct 16;151(3):407–14. doi: 10.1002/ijgo.13397 (PMC9087547; doi:10.1002/ijgo.13397)
Supplement: Supplementary file 1 — Table S1. Principal component analysis: rotated component matrix. [file IJGO-151-407-s001.pdf]

Supplementary Table 1. Principal Component Analysis: Rotated Component Matrix

	Component			
	1	2	3	4
I've been feeling down	**0.713**[a]	–0.086	–0.020	0.022
I've had trouble relaxing	**0.666**[a]	0.090	0.172	0.029
I've been feeling lonely	**0.618**[a]	0.051	0.162	0.088
I've been feeling worried	**0.531**[a]	0.043	–0.226	–0.147
I've become easily annoyed or irritable	**0.482**[a]	0.113	–0.010	–0.072
I've had time to exercise	–0.025	**0.697**[b]	0.069	0.176
I've had time to focus on my health	0.097	**0.670**[b]	-0.046	–0.051
I've had time to enjoy personal interests or hobbies	0.072	**0.579**[b]	0.089	–0.221
I've been having a poor appetite	–0.039	0.031	**0.733**[c]	–0.080
I've had trouble falling or staying asleep	0.339	0.017	**0.483**[c]	0.122
I feel connected with my local community	0.098	0.362	–0.404	–0.151
I've been overeating	0.071	0.250	0.327	–0.116
I've had the opportunity to chat with my family and friends	0.046	–0.255	–0.051	**0.609**[d]
I've enjoyed the spring weather	–0.055	0.020	0.327	**0.501**[d]
I've been feeling tired or having little energy	0.326	0.081	0.137	–0.489
I feel able to cope with the situation	0.310	0.208	–0.038	**0.442**[d]

Rotation Method: Varimax with Kaiser Normalization.

[a.] Component 1: Maternal mental health.

[b.] Component 2: Time availability for own health, hobbies, and interests.

[c.] Component 3: Appetite and sleep disruption.

[d.] Component 4: Coping.
